# Data to model the effect of awareness on the success of IT Governance implementation: A partial least squares structural equation modeling approach (PLS-SEM)

**DOI:** 10.1016/j.dib.2019.104333

**Published:** 2019-07-27

**Authors:** Uky Yudatama, Achmad Nizar Hidayanto, Bobby A.A. Nazief, Kongkiti Phusavat

**Affiliations:** a Faculty of Computer Science, Universitas Indonesia, Indonesia; b Kasetsart University, Thailand

**Keywords:** Awareness, Implementation, IT Governance, Structural equation modeling, Model

## Abstract

This article presents raw inferential statistical data that determine the influence of awareness on the successful implementation of IT Governance. Data were collected from respondents in all regions of Indonesia. Quantitative research methods are used to analyze data. The structured questionnaire was distributed to respondents in all regions of Indonesia who understood the field of IT Governance whose reliability and validity were confirmed. Structural equation modeling (SEM) using Smart PLS software, version 3, is used to present data. SEM path analysis shows an estimate of the relationship of the main constructs in the data. The results obtained from this dataset show a positive relationship between Risk Management, IT Resources, Budget, Stakeholder Involvement, Policy, Business Strategy, Organization, Commitment, Competence, Communication to awareness and consciousness also has a significant influence on the success of IT Governance implementation. However, politics has proven to have a negative and insignificant influence on the awareness and success of implementing IT Governance.

**Table:** Specifications Table

Subject area	*Business, Management and Accounting*
More specific subject area	*Organizational Behavior and Human Resource Management*
Type of data	*Tables and figures*
How data was acquired	*Data was collected significantly by distributing questionnaires to respondents who understood the field of IT Governance in all regions of Indonesia*
Data format	*Raw, analyzed, descriptive and statistical data*
Experimental factors	*The sample consisted of respondents who understood the field of IT Governance within the territory of Indonesia. The researcher has made a questionnaire that includes demographic data and research questions related to the variables being investigated and resolved.* *In this paper, Risk Management, IT Resources, Budget, Stakeholder Involvement, Policy, Business Strategy, Organization, Commitment, Politics, Competence, Communication have the influence on the awareness and success of implementing IT Governance have been studied.*
Experimental features	*Important variables in awareness such as Risk Management, IT Resources, Budget, Stakeholder Involvement, Policy, Business Strategy, Organization, Commitment, Politics, Competence, Communication play an important role awareness and in the success in implementing IT Governance.*
Data source location	*All regions of Indonesia*
Data accessibility	*Data are presented this article*

**Table:** 

**Value of the Data** • This data is useful because it can be used as a reference, input, and consideration in implementing IT Governance in order to experience success in accordance with organizational goals, namely the creation of harmony between business objectives and IT goals. • This data is useful for all parties involved, especially the top leaders of the organization, namely the board of directors and executive managers. • This data can be developed into a measurement tool to determine the extent of awareness of IT Governance in an organization. • The added value of this data is to provide a valuable contribution in the development of knowledge in the field of IT Governance, specifically soft IT Governance that concentrates on human behavior in its role to achieve successful implementation of IT Governance.

## 1 Data

**Fig. 1:**
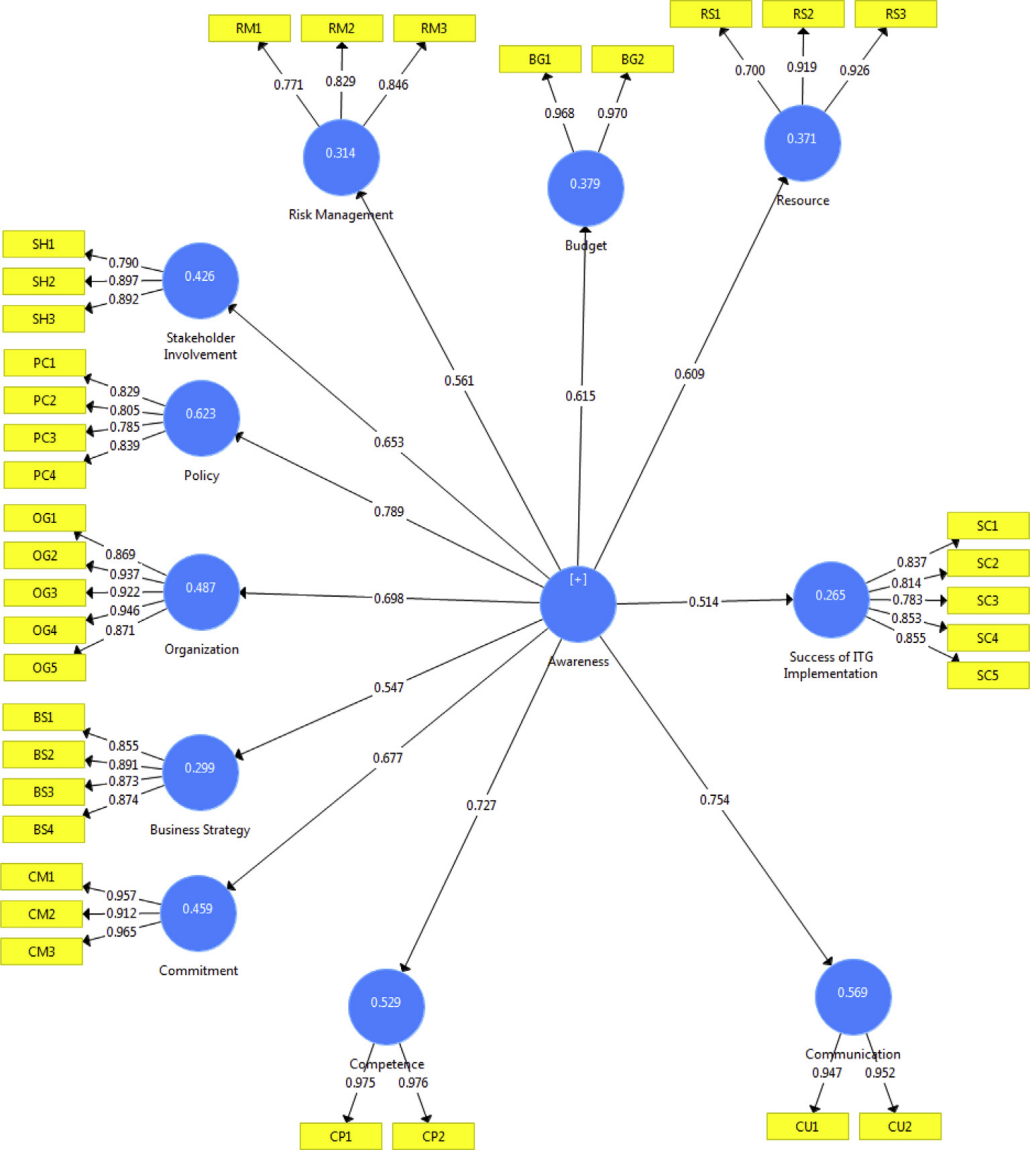
Measurement and structural model results.

Preliminary data were obtained through literature studies, as seen in Table 1. From the literature studies obtained later developed into a questionnaire. The questionnaire through an online survey can be accessed at the URL http://bit.ly/2XLNsPi, this questionnaire is then distributed to various communities that understand the field of IT Governance in all regions of Indonesia, as many as 260 copies of questionnaires are included, through a selection and feasibility process taken only 253 copy (97%). The survey questionnaire was chosen because it was considered the most preferred technique because of its many advantages and good quality [1]. To meet the quality feasibility, this data is then analyzed by considering the values: Cronbach's Alpha (0.6), Composite Reliability (0.7), AVE (0.5) and Loading Factor (0.7) [2], [3]. To determine the level of a significant path coefficient, the bootstrap and T-Statistic processes are used above 1.96 at the 95% confidence interval [4]. The measurement accuracy data can be seen in Table 2 and the structural model can be seen in Fig. 1. As the last data, Table 3 displays the output model analysis data.

**Table 1:** Variable in awareness IT Governance for implementation success.

Area	Sub Area
Risk Management **(RM)**	- Risk management related to the use and application of IT methodology **(RM1).** - Risk management related to control and supervision of IT resources **(RM2).** - Risk management of the strengths and weaknesses of IT related to evaluation and analysis **(RM3).**
IT Resources **(RS)**	- Resources for availability and fulfillment related to data, technology and applications **(RS1).** - Resources for management and supervision related to data, technology and applications **(RS2).** - Resources for portfolio management related to IT strategic assets **(RS3).**
Budget **(BG)**	- The budget for IT investments is related to size and ability **(BG1).** - Budget related to the availability of the IT budget needed **(BG2).**
Stakeholder involvement **(SH)**	- Stakeholder involvement in the implementation of IT Governance related to commissioners and board of directors **(SH1).** - Stakeholder involvement in the implementation of IT Governance related to executive managers **(SH2).**Stakeholder involvement in the implementation of IT Governance related to IT staff **(SH3).**
Policy **(PC)**	- Policies related to IT principles and responsibilities **(PC1).** - Policies on IT Governance rules and guidelines regarding decisions and compliance **(PC2).** - Policies related to the selection and use of best practices in IT Governance **(PC3).** - Policy towards alignment of integration between business and IT related to strategic decisions **(PC4).**
Business Strategy **(BS)**	- Business strategies related to alignment with business with IT **(BS1).** - Business strategy towards IT principles and policies related to decision making **(BS2).** - Business strategies related to IT monitoring of strategic changes **(BS3).** - Business strategies for control and supervision related to data, technology and applications **(BS4).**
Organization **(OG)**	- Organizations related to the atmosphere and climate conducive to organizational culture **(OG1).** - Organizations related to the performance of directors and executive managers on the existence of a supervisory board **(OG2).** - Organizations for environmental change related to adaptation **(OG3).** - Organization of regulations and external policies related to compliance **(OG4).** - Organizations related to the climate of empowerment and responsibility **(OG5).**
Commitment **(CM)**	- Commitments related to the decisions of the commissioners and the board of directors regarding rules and policies **(CM1).** - Commitments related to executive manager support for rules and policies **(CM2).** - Commitments related to IT staff compliance with rules and policies **(CM3).**
Politics **(PL)**	- Politics related to decision making **(PL1).** - Politics related to policy formulation **(PL2).** - Politics related to organizational elements **(PL3).**
Competence **(CP)**	- Competence towards mastery related to IT skills and skills **(CP1).** - Competence towards improving the quality of performance related to IT training and education **(CP2).**
Communication **(CU)**	- Communication with the board of directors and executive managers regarding feedback and two directions **(CU1).** - Communication with the board of directors and executive managers regarding the determination of strategy and expectations **(CU2).**
Success of IT Governance Implementation **(SC)**	- Improved Performance **(SC1).** - Provide added value **(SC2).** - Achieved alignment of objectives **(SC3).** - Risk Reduction **(SC4).** - Efficient and Effective **(SC5).**

**Table 2:** Measurement accuracy assessment.

Research Constructs	PLS code item	Scale item		Cronbach's Alpha value	Composite reliability	Average variance extracted (AVE)	Factor loading	P Values
		Mean	SD					
RM	RM1	0.512	0.059	0.750	0.857	0.666	0.772	0.000
	RM2	0.433	0.062				0.829	0.000
	RM3	0.412	0.072				0.845	0.000
RS	RS1	0.500	0.064	0.805	0.889	0.731	0.699	0.000
	RS2	0.523	0.064				0.919	0.000
	RS3	0.518	0.066				0.926	0.000
BG	BG1	0.583	0.060	0.935	0.969	0.939	0.968	0.000
	BG2	0.605	0.058				0.97	0.000
SH	SH1	0.489	0.070	0.825	0.896	0.742	0.79	0.000
	SH2	0.579	0.061				0.897	0.000
	SH3	0.608	0.050				0.892	
PC	PC1	0.647	0.055	0.831	0.888	0.664	0.829	0.000
	PC2	0.600	0.063				0.805	0.000
	PC3	0.610	0.062				0.785	0.000
	PC4	0.691	0.047				0.839	0.000
BS	BS1	0.462	0.059	0.896	0.928	0.763	0.855	0.000
	BS2	0.502	0.058				0.891	0.000
	BS3	0.447	0.063				0.873	0.000
	BS4	0.481	0.060				0.874	0.000
OG	OG1	0.634	0.046	0.947	0.960	0.827	0.869	0.000
	OG2	0.629	0.045				0.937	0.000
	OG3	0.624	0.050				0.922	0.000
	OG4	0.627	0.045				0.946	0.000
	OG5	0.646	0.044				0.871	0.000
CM	CM1	0.633	0.043	0.940	0.961	0.893	0.957	0.000
	CM2	0.646	0.042				0.912	0.000
	CM3	0.637	0.044				0.965	0.000
PL	PL1	−**0.058**	0.052	0.594	0.784	0.548	0.721	**0.270**
	PL2	−**0.076**	0.060				0.776	**0.177**
	PL3	−**0.065**	0.052				0.722	**0.201**
CP	CP1	0.698	0.039	0.949	0.975	0.952	0.975	0.000
	CP2	0.722	0.034				0.976	0.000
CU	CU1	0.697	0.039	0.892	0.949	0.902	0.947	0.000
	CU2	0.734	0.033				0.952	0.000
SC	SC1	0.837	0.026	0.886	0.916	0.687	0.837	0.000
	SC2	0.810	0.033				0.814	0.000
	SC3	0.781	0.033				0.783	0.000
	SC4	0.850	0.028				0.853	0.000
	SC5	0.855	0.024				0.855	0.000

Bold values indicates Special attention/Not eligible.

**Table 3:** Outcomes of structural equation model analysis.

Path	Hypothesis	Path coefficient (β)	T-Statistics	Decision
Awareness -> Risk Management	H1 (+)	0.560	9.837	Positive and significant
Awareness -> Resource	H2 (+)	0.609	11.415	Positive and significant
Awareness -> Budget	H3 (+)	0.615	10.449	Positive and significant
Awareness -> Stakeholder Involvement	H4 (+)	0.653	11.568	Positive and significant
Awareness -> Policy	H5 (+)	0.789	23.855	Positive and significant
Awareness -> Business Strategy	H6 (+)	0.547	9.613	Positive and significant
Awareness -> Organization	H7 (+)	0.698	16.328	Positive and significant
Awareness -> Commitment	H8 (+)	0.677	16.477	Positive and significant
Awareness -> Politics	H9 (+)	−**0.094**	**0.804**	**Negative and insignificant**
Awareness -> Competence	H10 (+)	0.727	21.046	Positive and significant
Awareness -> Communication	H11 (+)	0.754	22.412	Positive and significant
Awareness -> Success of IT Governance Implementation	H12 (+)	0.515	7.715	Positive and significant

Bold values indicates Special attention/Not eligible.

## 2 Experimental design, materials, and methods

The data presented is based on qualitative and quantitative research. Qualitative data were obtained based on literature studies to obtain awareness variables, as seen in Table 1. While quantitative data were obtained by distributing questionnaires to respondents. The survey method is considered as the right data collection method because it enables standardized data collection that allows researchers to produce information answering important variable questions that influence the awareness and success in implementing IT Governance. Respondents in the Indonesian country were selected for this study. To test the data, researchers propose a model where Risk Management, IT Resources, Budget, Stakeholder Involvement, Policy, Business Strategy, Organization, Commitment, Politics, Competence, Communication are outcome variables. The model proposed by the researcher must be tested for validity from the proposed model and to determine whether the data, which has been collected in the field, matches the proposed conceptual model. The quality of the measurement model is determined based on its validity and reliability [2], [3]. The results of testing the validity and reliability of the data are shown in Table 2.

### 2.1 Path model

The PLS estimation results for the structural model, path coefficients values as well as the item loadings for the research constructs are shown in Fig. 1 (Table 3).

The main data source (questionnaire) is used to collect data from respondents in the territory of Indonesia. The Microsoft Excel spreadsheet worksheet is used to enter all data and draw conclusions from the data obtained. The Statistical Package for Social Sciences (SPSS) and Smart PLS software for structural equation modeling techniques (SEM) is used to record data and carry out the statistical analysis. In addition, Smart PLS supports exploratory and confirmation research; normal multivariate and good for small sample sizes.

### 2.2 Ethical considerations

The researcher guarantees that the respondents have adequate knowledge related to the purpose of this research, besides that they also obtain complete and transparent information. Respondents are guaranteed confidentiality about their personal data.

### 2.3 Academic, practical, and policy implications of this data article

The data presented in this article have implications for academics, for example, awareness directly influences the success of implementing IT governance in a positive and significant way as indicated by the path coefficient of (β = 0.515).

Therefore, for academics in the field of IT Governance, this discovery can enhance their understanding of the relationship between awareness and success of IT Governance. This is a useful contribution to be used as literature. On the practitioner's side, the board of directors and executive managers can benefit from the implications of this discovery. For example, there is a strong relationship between awareness and risk management (β = 0.560), executive managers must pay attention to risk management related to the use of methodology, monitoring of IT resources and evaluating weaknesses and strengths [5], [6]. In addition, this data article offers implications for policymakers (board of directors) for the implementation of IT governance in order to improve company performance by paying attention to variables in consciousness. Thus, findings obtained from this research data collection can be used to generate new policies and assist in revising existing policies.
