# Supplementary material for: Data to model the effect of awareness on the success of IT Governance implementation: A partial least squares structural equation modeling approach (PLS-SEM)
Source: Data Brief. 2019 Jul 27;25:104333. doi: 10.1016/j.dib.2019.104333 (PMC6717165; doi:10.1016/j.dib.2019.104333)
Supplement: Multimedia component 1 [file mmc1.docx]

*Questionnaire Form*

<http://bit.ly/2XLNsPi>

**EFFECT OF AWARENESS ON THE SUCCESS OF IT GOVERNANCE IMPLEMENTATION**

*This questionnaire aims to find out important variables that can influence awareness of the success of IT Governance Implementation*

1. **RISK MANAGEMENT**

*Risk is all possible events that can harm the company*

1. Is risk management related to the use and application of IT methodologies needed in the implementation of IT Governance?

a. Strongly Disagree

b. Disagree

c. Undecided

d. Agree

e. Strongly Agree

1. Is risk management related to the control and supervision of IT resources needed in implementing IT Governance?

a. Strongly Disagree

b. Disagree

c. Undecided

d. Agree

e. Strongly Agree

1. Is risk management from the strengths and weaknesses of IT related to evaluation and analysis needed in the implementation of IT Governance?

a. Strongly Disagree

b. Disagree

c. Undecided

d. Agree

e. Strongly Agree

1. **IT RESOURCES**

*IT Resources are all means related to IT that can support an organization's business processes.*

1. Are resources for availability and fulfillment related to data, technology, and applications needed in the implementation of IT Governance?

a. Strongly Disagree

b. Disagree

c. Undecided

d. Agree

e. Strongly Agree

1. Are resources related to data, technology, and applications required supervision and management?

a. Strongly Disagree

b. Disagree

c. Undecided

d. Agree

e. Strongly Agree

1. Are resources related to IT strategic assets for portfolio management needed in the implementation of IT Governance?

a. Strongly Disagree

b. Disagree

c. Undecided

d. Agree

e. Strongly Agree

1. **BUDGET**

*The budget is a periodic financial plan that is prepared based on the approved program.*

1. Is the budget for IT investment in implementing IT Governance tailored to the size and capability of the organization?

a. Strongly Disagree

b. Disagree

c. Undecided

d. Agree

e. Strongly Agree

1. Is the budget needed IT in the implementation of IT Governance adjusted to availability?

a. Strongly Disagree

b. Disagree

c. Undecided

d. Agree

e. Strongly Agree

1. **STAKEHOLDER**

*Stakeholder involvement is a participatory process by empowering all parties to achieve certain goals.*

1. Is the involvement of the board of directors and commissioners needed in the implementation of IT Governance?

a. Strongly Disagree

b. Disagree

c. Undecided

d. Agree

e. Strongly Agree

1. Is the involvement of executive managers needed in implementing IT governance?

a. Strongly Disagree

b. Disagree

c. Undecided

d. Agree

e. Strongly Agree

1. Is the involvement of IT staff needed in implementing IT governance?

a. Strongly Disagree

b. Disagree

c. Undecided

d. Agree

e. Strongly Agree

1. **POLICIES**

*The policy is the decision of an organization to overcome certain problems as a decision or to achieve certain goals, containing provisions that can be used as guidelines.*

1. Are policies related to IT principles and responsibilities needed in the implementation of IT Governance?

a. Strongly Disagree

b. Disagree

c. Undecided

d. Agree

e. Strongly Agree

1. Are policies related to decisions and compliance with IT Governance rules and guidelines needed in the implementation of IT Governance?

a. Strongly Disagree

b. Disagree

c. Undecided

d. Agree

e. Strongly Agree

1. Are policies related to the selection and use of best practices needed in the implementation of IT Governance?

a. Strongly Disagree

b. Disagree

c. Undecided

d. Agree

e. Strongly Agree

1. Is the policy to alignment business-IT integration needed in the implementation of IT Governance?

a. Strongly Disagree

b. Disagree

c. Undecided

d. Agree

e. Strongly Agree

1. **BUSINESS STRATEGY**

*Business strategy is the way organizations associate their activities into the business direction.*

1. Is a business strategy also needed to achieve harmony between business goals and IT goals?

a. Strongly Disagree

b. Disagree

c. Undecided

d. Agree

e. Strongly Agree

1. Does the business strategy regarding IT principles and policies related to decision making can determine the success of IT Governance implementation?

a. Strongly Disagree

b. Disagree

c. Undecided

d. Agree

e. Strongly Agree

1. Does a business strategy related to IT monitoring of strategic change affect the success of IT Governance implementation?

a. Strongly Disagree

b. Disagree

c. Undecided

d. Agree

e. Strongly Agree

1. Are controls and supervision related to data, technology, and applications a business strategy needed in implementing IT Governance?

a. Strongly Disagree

b. Disagree

c. Undecided

d. Agree

e. Strongly Agree

1. **ORGANIZATION**

*Organizations are a group of people in a forum to realize a common goal.*

1. Is an organization with an atmosphere and a conducive climate needed in the implementation of IT Governance?

a. Strongly Disagree

b. Disagree

c. Undecided

d. Agree

e. Strongly Agree

1. Is the existence of a supervisory board in the Organization related to the performance of directors and executive managers needed in the implementation of IT Governance?

a. Strongly Disagree

b. Disagree

c. Undecided

d. Agree

e. Strongly Agree

1. Are environmental changes to organizations related to adaptation needed in the implementation of IT Governance?

a. Strongly Disagree

b. Disagree

c. Undecided

d. Agree

e. Strongly Agree

1. Are external regulations and policies in the organization related to compliance required in the implementation of IT Governance?

a. Strongly Disagree

b. Disagree

c. Undecided

d. Agree

e. Strongly Agree

1. Is the climate of empowerment and responsibility within the organization needed in the implementation of IT Governance?

a. Strongly Disagree

b. Disagree

c. Undecided

d. Agree

e. Strongly Agree

1. **COMMITMENT**

*Commitment is a situation where someone makes an agreement, both to themselves and to others, which is reflected in certain actions/behaviors carried out voluntarily or forcibly.*

1. Is the commitment to the decision of the commissioner and the board of directors regarding regulations and policies needed in implementing IT Governance?

a. Strongly Disagree

b. Disagree

c. Undecided

d. Agree

e. Strongly Agree

1. Are commitments related to manager executive support for regulations and policies needed in the implementation of IT Governance?

a. Strongly Disagree

b. Disagree

c. Undecided

d. Agree

e. Strongly Agree

1. Are commitments related to IT staff compliance with regulations and policies needed in the implementation of IT Governance?

a. Strongly Disagree

b. Disagree

c. Undecided

d. Agree

e. Strongly Agree

1. **POLITICS**

*Politics is a process of decision making in an effort to gain power both legally and non-legally.*

1. Is politics related to decision making needed in the implementation of IT Governance?

a. Strongly Disagree

b. Disagree

c. Undecided

d. Agree

e. Strongly Agree

1. Is politics related to policy formulation needed in the implementation of IT Governance?

a. Strongly Disagree

b. Disagree

c. Undecided

d. Agree

e. Strongly Agree

1. Is politics related to organizational elements needed in the implementation of IT Governance?

a. Strongly Disagree

b. Disagree

c. Undecided

d. Agree

e. Strongly Agree

1. **COMPETENCE**

*Competence is a set of knowledge, skills, attitudes, and values as a performance that affects one's role, actions, achievements, and work.*

1. Are competencies related to mastering IT skills and skills needed in the implementation of IT Governance?

a. Strongly Disagree

b. Disagree

c. Undecided

d. Agree

e. Strongly Agree

1. Are competencies about improving the quality of performance related to IT training and education needed in the implementation of IT Governance?

a. Strongly Disagree

b. Disagree

c. Undecided

d. Agree

e. Strongly Agree

1. **COMMUNICATION**

*Communication is a process of delivering information (messages or ideas) from one party to another****.***

1. Are feedback and two-way communication between the board of directors and executive managers needed in the implementation of IT Governance?

a. Strongly Disagree

b. Disagree

c. Undecided

d. Agree

e. Strongly Agree

1. Is communication between the board of directors and executive managers regarding the determination of strategies and expectations needed in the implementation of IT Governance?

a. Strongly Disagree

b. Disagree

c. Undecided

d. Agree

e. Strongly Agree

1. ***IT GOVERNANCE SUCCESS***

*The success of IT Governance is something that can be achieved according to what is expected and can provide benefits and value add for the organization.*

1. Does the implementation of IT Governance can improve performance?

a. Strongly Disagree

b. Disagree

c. Undecided

d. Agree

e. Strongly Agree

1. Does the implementation of IT Governance can provide added value?

a. Strongly Disagree

b. Disagree

c. Undecided

d. Agree

e. Strongly Agree

1. Does the implementation of IT Governance can achieve the alignment of organizational goals?

a. Strongly Disagree

b. Disagree

c. Undecided

d. Agree

e. Strongly Agree

1. Does the implementation of IT Governance can reduce risk?

a. Strongly Disagree

b. Disagree

c. Undecided

d. Agree

e. Strongly Agree

1. Does the implementation of IT Governance can achieve Efficiency and Effectiveness?

a. Strongly Disagree

b. Disagree

c. Undecided

d. Agree

e. Strongly Agree

*Thank you*

*I really appreciate your time and participation in this questionnaire.*

*Have a nice day …*

Best regards,

**Uky Yudatama**
